# Voxel-based morphometric imaging in first-episode psychosis: interrogating the role of familial liability

**DOI:** 10.1192/j.eurpsy.2023.1158

**Published:** 2023-07-19

**Authors:** F. C. Corsi-Zuelli, F. L. Souza Duran, C. M. Loureiro, A. C. dos Santos, G. Busatto, P. R. Menezes, C. M. Del-Ben

**Affiliations:** 1 Neuroscience, University of São Paulo, Ribeirão Preto Medical School, Ribeirao Preto; 2 Department and Institute of Psychiatry, Faculty of Medicine, University of São Paulo, Brazil, São Paulo; 3 Neuroradiology, University of São Paulo, Ribeirão Preto Medical School, Ribeirao Preto; 4 Preventive Medicine, Faculty of Medicine, University of São Paulo, Brazil, São Paulo, Brazil

## Abstract

**Introduction:**

Neuroanatomical abnormalities are reported in psychotic disorders compared to healthy controls; nevertheless, less is known about the role of familial liability to psychosis in morphological brain changes.

**Objectives:**

Using an exploratory voxel-based morphometry (VBM) analyses of the whole brain, we evaluated differences on GMVs across the whole brain among first-episode psychosis (FEP) patients, community-controls, and healthy siblings of patients to interrogate the role of familial liability.

**Methods:**

Data were retrieved from a study (STREAM) conducted in Ribeirão Preto/SP Brazil. We included 71 first-episode psychosis patients (67.6% males, mean age±SD: 18.7±10.8), 24 unaffected siblings of patients (37.5% males, mean age±SD 30.8±10), and 36 controls (71.9% males, mean age±SD: 10±10.5). All magnetic resonance imaging (MRI) scans were acquired on a 3T Philips scanner. VBM data were processed using Statistical Parametric Mapping (SPM) software in MATLAB the MNI coordinate system. We performed exploratory voxel-wise comparisons of GMVs among the three groups using an analysis of covariance (ANCOVA) model in SPM. Results were considered significant if they retained significance after family-wise error (FWE) correction for multiple comparisons (p<0.05). All the analyses were adjusted for age, sex, education in years, and total brain GMV.

**Results:**

The whole-brain exploratory analyses revealed no significant findings at the p<0.05 level (FWE-corrected). However, pairwise comparisons revealed significant changes betweeen FEP patients and their unaffected siblings. In particular, FEP patients had decreased volumes in the right side of the following regions (FEW = 0.047): superior temporal cortex, Rolandic operculum, insula, Heschel’s gyrus, supramarginal gyrus, superior temporal pole, hippocampus, parahippocampal gyrus, fusiform gyrus, amydgala, olfactory, inferior frontal operculum, cerebellum, posterior and medial orbital frontal cortex, rectus, medial temporal, medial frontal, and putamen. FEP patients also showed decreased volumes on the left side of the following regions (FWE 0.049): frontal superior medial gyrus, superior frontal gyrus, frontal middle part, caudate, anterior cingulate cortex, thalamus, and pallidum. Patients also showed widespread reduced GMV in various GMVs regions compared to controls at FWE<0.05. However, no difference was found between siblings and controls (FWE: >0.05).

**Conclusions:**

The study of healthy siblings of patients with heritable illnesses could help in the understanding of the contribution of genetic background and environmental factors to illness state and predisposition. Differences between patients and their siblings could be attributed to the disease state, considering that the unaffected sibling group and unrelated healthy control group did not differ. We will next evaluate biological and environmental contributors to the reported differences.

**Disclosure of Interest:**

None Declared

